# Influence and molecular mechanism of cinnamaldehyde against ventricular arrhythmia via the TAK1-p38MAPK-NLRP3 pathway

**DOI:** 10.1007/s00380-025-02529-3

**Published:** 2025-03-27

**Authors:** Guoping Ma, Mian Li, Wanyue Yang, Hai Wang, Xue Tian, Yajuan Yin, Yida Tang, Wenjie Liang

**Affiliations:** 1 The First Hospital of Hebei Medical University, Shijiazhuang, 050031 China; 2 The Third Hospital of Hebei Medical University, Shijiazhuang, 050051 China; 3 Department of Cardiology, Institute of Vascular Medicine, State Key Laboratory of Vascular Homeostasis and Remodelling, NHC Key Laboratory of Cardiovascular Molecular Biology and Regulatory Peptides, Key Laboratory Ofcardiovascular Receptors Research, Peking University Third Hospital, Peking University, BeijingBeijing, 100191 China; 4 Hebei University of Chinese Medicine, Shijiazhuang, 050200 China; 5 Wuan Hospital of traditional Chinese Medicine, Wuan, 056300 China

**Keywords:** Ventricular arrhythmia, Cinnamaldehyde, Transforming growth factor-β activated kinase 1, P38 mitogen-activated protein kinase, Nucleotide binding oligomeric domain-like receptor protein 3

## Abstract

Based on the transforming growth factor β-activated kinase 1 (TAK1)-p38 mitogen-activated protein kinase (p38MAPK)-nucleotide-binding oligo-like receptor protein 3 (NLRP3) signalling pathway, the protective effect and mechanism of isoproterennaline (ISO)-induced cinnamaldehyde on inflammatory injury in ventricular rats were investigated. Fifty male SPF SD rats were randomly assigned to the normal group, model group, propranolol group, cinnamaldehyde low-dose group or cinnamaldehyde high-dose group. The ventricular arrhythmia model was constructed using the "6 + 1" ISO injection method. The rats in the propranolol group were given propranolol 15 mg·(kg d)^−1^, those in the low and high-dose groups were given cinnamaldehyde 20 mg·(kg d)^−1^ and 50 mg·(kg d)^−1^, respectively, and those in the control and model groups received an equal volume of 0.9% NaCl solution. Changes in the serum troponin (cTnI), creatine kinase isoenzyme (CK-MB), and interleukin-1β (IL-1β) levels in SD rats were determined by ELISA. HE staining was used to observe the tissue morphology of heart disease. The mRNA expression of IL-1β and NLRP3 was determined by RT‒PCR. Mitochondrial damage was observed by transmission electron microscopy. The expression of reactive oxygen species (ROS) was detected by immunofluorescence. Western blot or immunohistochemical detection of the protein expression of IL-1β, NLRP3, TAK1, phospho-TAK1 (p-TAK1), p38MAPK, phospho-p38MAPK (p-p38MAPK), nuclear factor-κB (NF-κB),and phospho-NF-κB (p-NF-κB) was also performed. Data analysis was performed using SPSS 25.0 software. In the control SD rats, there were no obvious ventricular arrhythmias on ECG, the cardiac tissue and mitochondria were basically normal, the serum IL-1β level was low, and the expression of myocardial IL-1β, NLRP3, ROS, p-TAK1, p-p38MAPK and p-NF-κB was weak. Compared with the control group, the model group of SD rats had significant increases in ventricular arrhythmia and arrhythmia scores according to ECG (*P* < 0.01). Myocardial histopathological injury, cardiac weight index (HWI) and increases in serum cTnI and CK-MB levels were detected (*P* < 0.01). Additionally, mitochondrial damage in myocardial tissue, increased ROS fluorescence intensity, and elevated expression of myocardial p-TAK1, p-p38MAPK and p-NF-κB were detected(*P* < 0.01). The protein and mRNA expression of inflammation-related factors NLRP3 and IL-1β were increased (*P* < 0.01 or *P* < 0.05). Compared with those in the model group, the arrhythmia scores were decreased in the three treatment groups (*P* < 0.01 or *P* < 0.05). Cardiac histopathological morphology was significantly improved, and HWI and myocardial injury-related indicators were decreased(*P* < 0.01 or *P* < 0.05). Damaged mitochondria were significantly improved, and the expression of ROS, p-TAK1, p-p38MAPK, and p-NF-κB were decreased. The expression of inflammation-related factors in serum and myocardial tissue was decreased (*P* < 0.01 or *P* < 0.05). TAK1-p38MAPK-NLRP3 signalling is enhanced in SD rats with ventricular arrhythmia. Cinnamaldehyde can regulate TAK1-p38MAPK-NLRP3 signalling, reduce cardiomyocyte pyroptosis, antagonize myocardial inflammatory injury and protect cardiomyocytes by inhibiting oxidative stress.

## Preface

Cardiovascular diseases (CVDs) are a class of noncommunicable diseases that are widely prevalent worldwide and have very high incidence and fatality rates. Epidemiology shows that there are more than 300 million CVD patients in China who often die from sudden cardiac death, and ventricular arrhythmia is a serious complication of CVD that can lead to cardiac haemodynamic changes and increase the risk of sudden death [1–3]. Therefore, early identification and timely intervention of ventricular arrhythmias are particularly important to reduce the incidence of adverse cardiovascular events.

The pathogenesis of ventricular arrhythmia is complex, it easily recurs, and specific drugs to block its progression are lacking in modern medicine. A previous study revealed that the release of oxidative stress and inflammation-related factors increased in an ISO-induced ischaemic arrhythmia model, indicating that oxidative stress and inflammation may be the two key factors involved in promoting the development of ventricular arrhythmias [4]. Transforming growth factor-β-activated kinase 1 (TAK1) is a key molecule in the inflammatory signalling pathway [5]. Oxidative stress can initiate TAK1 activation followed by activation of p38 mitogen-activated protein kinase (p38MAPK) via a tertiary phosphorylation cascade; the latter acts on nuclear factor-κB (NF-κB) and other transcription factors, regulates nucleotide binding oligomeric domain-like receptor protein 3 (NLRP3) expression and promotes the inflammatory response [6, 7]. However, few studies on TAK1 signalling in ventricular arrhythmia patients have been reported.

Cinnamaldehyde is a class of natural active substances isolated from the plant cinnamon that has anti-inflammatory, antioxidant and antiapoptotic pharmacological effects [8, 9]. Previous studies have shown that cinnamaldehyde downregulates inflammatory gene expression and reduces myocardial inflammatory injury [10] in a myocardial ischaemia–reperfusion model by inhibiting NLRP3 activation. In fructose-induced myocarditis, cinnamaldehyde also improves cardiac function [11] by regulating NLRP3 expression. Cinnamaldehyde can exert protective effects against a variety of CVDs, but the exact mechanism remains unclear. In a previous study by our team, cinnamaldehyde significantly improved ISO-induced acute myocardial injury and postischemic ventricular arrhythmia, which is in line with the results reported by Song F and Mehrnoosh et al. [12, 13] Based on previous research, this paper focused on the mechanism of ventricular arrhythmia and the effect of cinnamaldehyde on the TAK1-p38MAPK-NLRP3 signalling pathway from the perspective of inflammation to provide new ideas and a basis for clinical treatment and new drug research and development.

## Materials and methods

### Animals

Fifty SPF healthy male 6-week-old SD rats with a weight of 80–100 g were purchased from Liaoning Changsheng Biotechnology Co., Ltd.(Experimental animal production certificate number: SCXK(Liao) 2020-0001). The experimental animals were kept in the central laboratory of the First Hospital of Hebei Medical University.

### Drugs

ISO(batch number: I5627) was purchased from Sigma-Aldrich Company. Propranolol tablets (batch number: E181234) were purchased from Jiangsu Yabang Pharmaceutical Group Co.,Ltd. Cinnamaldehyde(batch number: IC1140) was purchased from Beijing Solaibao Technology Co.,Ltd.

### Reagent

Antibodies against TAK1, phospho-TAK1 (p-TAK1), p38MAPK, and phospho-p38MAPK(p-p38MAPK) (batch number: 5206S, 9339S, 8690S, 4511T) were purchased from Cell Signaling Technology Company. Nuclear factor-κB (NF-κB) antibody (lot number: 10745-1-AP) was purchased from Proteintech Group Company. phospho-NF-κB (p-NF-κB) antibody and interleukin-1β (IL-1β) antibody (batchnumber: AF2006, AF5103, AF7014) and NLRP3 antibody(batchnumber: ET1610-93) were purchased from Hua'an Biotechnology Co.,Ltd. Reactive oxygen species(ROS) dye solution(lot number: D7008) was purchased from Sigma-Aldrich.

### Animal grouping, model preparation, and drug intervention

Fifty SD rats were fed adaptively for one week and were evenly divided into 5 groups according to the random number table method: the control group, model group, propranolol group, and low and high-dose cinnamaldehyde groups, with 10 rats in each group. Guo et al. [14] used a "6 + 1" injection of isoproterenol (ISO) to induce ventricular arrhythmia in an SD rat model. Rats were gavaged 1 time/d for 7 d. Propranolol 15 mg·(kg d)^−1^ was administered to the propranolol group [4]. It is reported that cinnamaldehyde can improve the cardiac injury caused by isoproterenol at the doses of 22.5 mg·(kg d)^−1^, 45 mg·(kg d)^−1^ and 90 mg·(kg d)^−1^ in rats by inhibiting the cardiac inflammatory reaction [12]. In addition, Lan et al. also reported that continuous gavage of 40 mg·(kg d)^−1^cinnamaldehyde for one week could improve the myocardial ischemia–reperfusion injury [15]. Therefore, the low and high-dose groups of cinnamaldehyde were given cinnamaldehyde 20 mg·(kg d)^−1^ and 50 mg·(kg d)^−1^, respectively.

### ECG observation and recording

The BL-420F biological function experimental system was used to monitor the changes in the standard lead ECG in SD rats before and after ISO induction, and then 2 or more relevant professionals were invited to interpret the ECG results and calculate the arrhythmia score. According to previous studies [4, 16], the determination and scoring criteria for arrhythmia are shown in Table 1.

**Table 1:** Arrhythmia scoring criteria

Score	Electrocardiographic features
0	Normal sinus rhythm
1	A large, malformed QRS wave group that appears early, that is,premature ventricular contraction(PVC)
2	Two or three consecutive PVC
3	4 ≤ The number of consecutive ventricular complex waves < 15, that is non-sustained ventricular tachycardia(NSVT)
4	The number of consecutive ventricular complex waves ≥ 15, that is sustained ventricular tachycardia(SVT)
5	Death

### Heart weight index test

The whole heart tissue of the SD rats was placed on ice, washed with saline and blotted dry with filter paper, and the heart weight of each group of SD rats was accurately measured. The cardiac weight index (HWI) was calculated as heart weight (HW) /body mass (BM).

### Detection of serum indicators

Femoral artery blood from SD rats was collected at room temperature for 1 h and then centrifuged at 4 °C and 4000 r min^−1^ for 15 min, after which the serum was retained. The following indicators were tested: serum cardiac troponin-I (cTnI), creatine kinase isoenzyme (CK-MB), and IL-1β.

### Morphological observations of cardiac disease

After the heart was weighed, half of the tissue was fixed with 4% paraformaldehyde to observe pathological morphology, and half was frozen at −80 °C for analysis of other indicators. The myocardial tissues were fixed with 4% paraformaldehyde for more than 24 h, dehydrated with an ethanol gradient, homogenized in paraffin, divided and dewaxed, and the morphological changes in the SD rats were detected by haematoxylin–eosin (HE) staining.

### Real-time PCR

The mRNA expression of IL-1β and NLRP3 was detected in the cardiac tissues of SD rats. Fifty milligrams of frozen myocardial tissue was harvested, and RNA was extracted and reverse transcribed into cDNA. The following 20 µl reaction mixture was used for amplification: 95 °C predenaturation for 30 s; 95 °C for 10 s; and 60 °C for 30 s for 40 cycles. The CT values of the target gene and reference gene of each group were obtained. ΔCT = CT value of target gene-CT value of reference gene, ΔCT = mean of ΔCT-control group ΔCT of each sample of the experimental group, and the relative expression value of the target gene in each group was 2^−ΔΔCT^. The primers were synthesized by Wuhan Sevwell Biotechnology Co., Ltd., and the primer sequences are listed in Table 2.

**Table 2:** Real Time-PCR primer sequence

Gene name	Primer sequence(5′−3′)	Length(bp)
NLRP3	forward primer: AGACCTCCAAGACCACGACTG	98
	reverse primer: TTCCATCCGCAGCCAATGAAC	
IL-1β	forward primer: TGTGACTCGTGGGATGATGAC	160
	reverse primer: CCACTTGTTGGCTTATGTTCTGTC	
GAPDH	forward primer: CTGGAGAAACCTGCCAAGTATG	138
	reverse primer: GGTGGAAGAATGGGAGTTGCT	

#### Observation by transmission electron microscopy

Fresh myocardial tissue(1 mm^3^ in size) was prefixed with 3% glutaraldehyde, fixed in 1% osmic acid for 2 h protected from light, dehydrated in ethanol, infiltrated, embedded, polymerized in a 60 °C oven for 48 h, sliced in an ultrathin slicing machine, and stained with 2% uranium acetate and 2.6% lead citrate. Then, we observed the mitochondrial structure of the SD rats and acquired images under a transmission electron microscope.

#### Immunofluorescence

ROS levels were detected in rat heart tissue. The myocardial tissue sections were frozen, rewarmed and drained at room temperature, after which a spontaneous fluorescent quenching agent was added, and the sections were rinsed with running water for 5 min for 10 min. ROS dye solution was added, and the sections were incubated in a 37 °C constant-temperature water bath for 30 min. The sections were washed with PBS shaking 3 times for 5 min/wash. After the slices were dried, DAPI dye solution was added, and the sections were incubated at room temperature for 10 min. The sections were washed 3 times, the anti-fluorescence quenching agent was added, and the images were collected under a fluorescence microscope.

#### The SABC method

The protein expression of NLRP3, IL-1β, p-TAK1, p-p38MAPK, and p-NF-κB was detected in the heart tissues of SD rats. According to previous literature [18], protein localization and expression were observed under a microscope, and the presence of brown particles indicated positive expression.

#### Western blot

The expression of NLRP3, IL-1β, TAK1, p-TAK1, p-p38MAPK, NF-κB, and p-NF-κB was detected in the cardiac tissues of SD rats. According to previous literature [18], the target protein grey values were calculated using ImageJ software, and the results were corrected with internal parameters.

#### Statistical analysis

Statistical analysis of the data was performed using SPSS 25.0 statistical software. The experimental data are expressed as the mean ± standard deviation ($\overline{x} \pm s$). If the data met the assumptions of normality and homogeneity of variance, the one-way variance was used for comparisons between multiple groups, and the LSD-*t* test was used for pairwise comparisons between groups. If the data did not meet the normal distribution or homogeneity of variance, a nonparametric test was used. Differences were considered statistically significant when *P* < 0.05.

## Results

### Comparison of ECGs of the rats in each group

#### Incidence of cardiac arrhythmia

**Fig.1:**
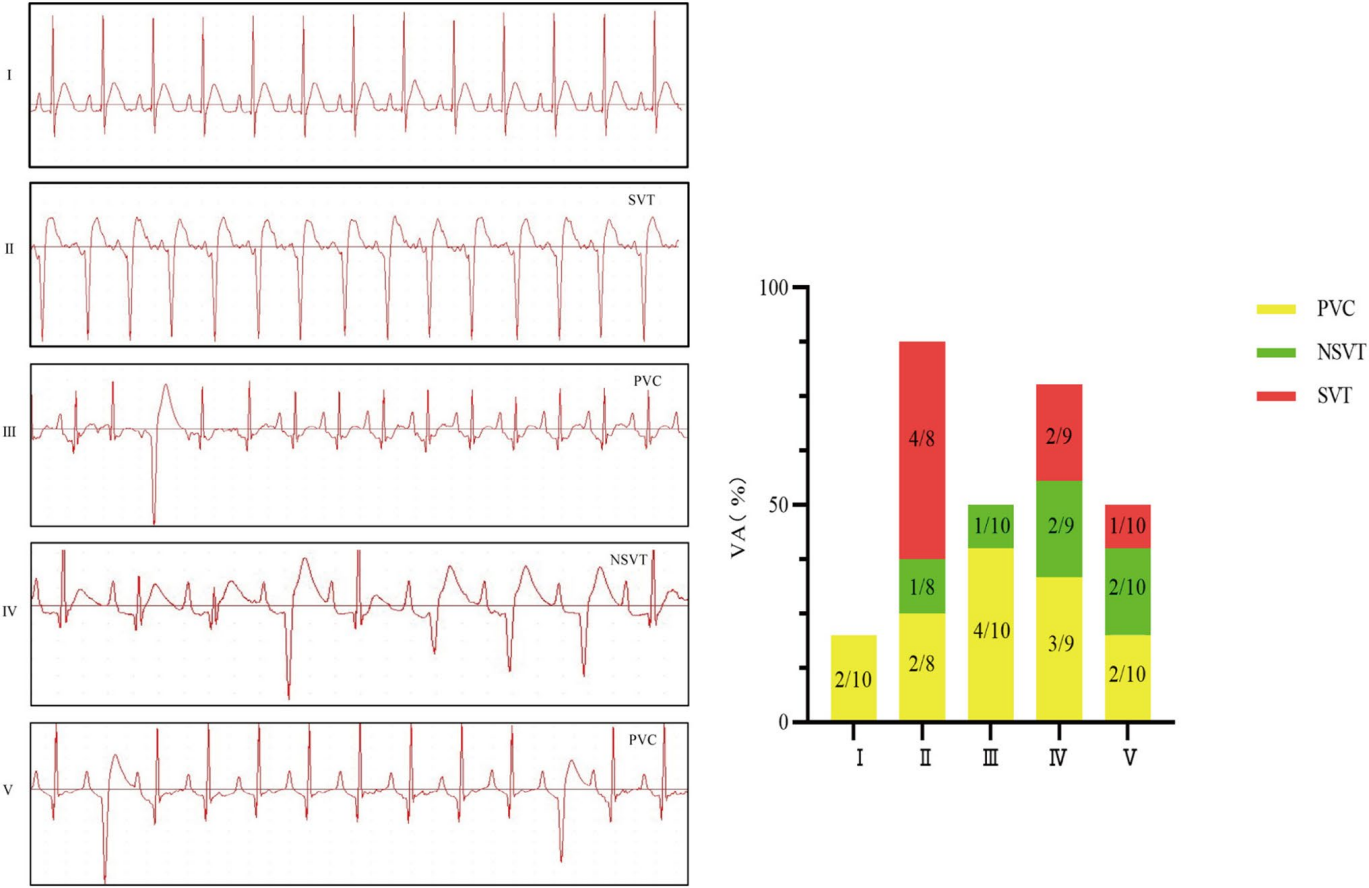
Occurrence of ventricular arrhythmia in each group. I Control group, II Model group, III Propranolol group, IV Cinnamaldehyde low dose group, V Cinnamaldehyde high-dose group

The ECG results showed that the ECGs of the control group were almost normal. The ECGs of the model group of rats that developed ventricular arrhythmia were mainly composed of SVT. Compared with those of the model group, SVT was reduced in each treatment group, and SVT reduction was more pronounced in the propranolol and cinnamon aldehyde high-dose groups. See Fig. 1.

#### Arrhythmia score

**Fig. 2:**
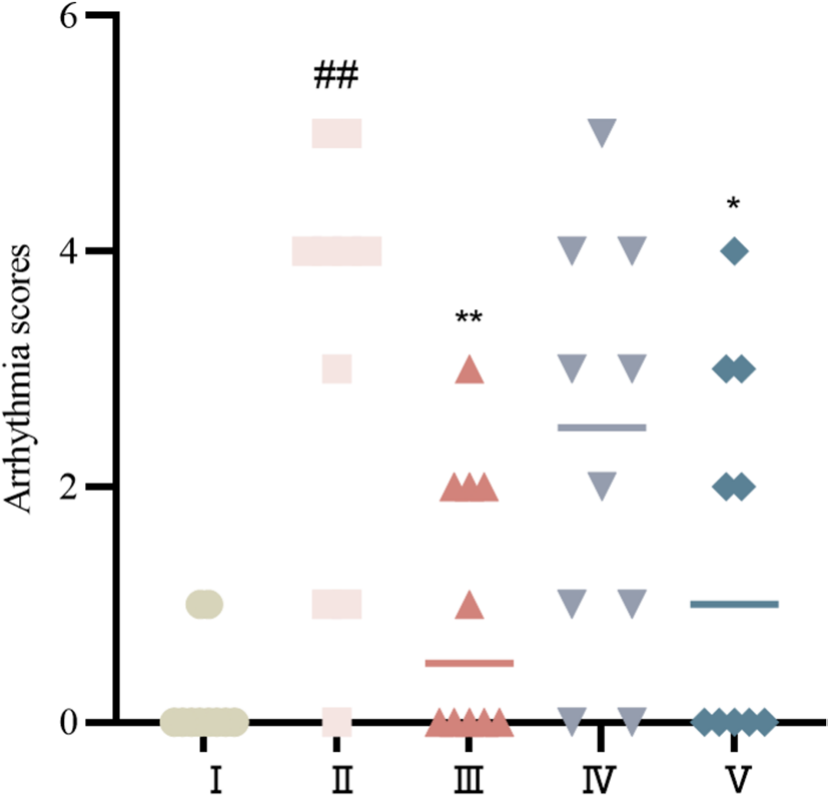
Arrhythmia score of rats in each group. I Control group, II Model group, III Propranolol group, IV Cinnamaldehyde low dose group, V Cinnamaldehyde high-dose group. Note: vs Control group, ^##^*P* < 0.01; vs Model group, ^*^P < 0.05, ^**^P < 0.01

Compared with those in the control group, the arrhythmia scores in the model group were significantly greater (*P* < 0.01). Compared with those in the model group, the arrhythmia scores in the propranolol group and the cinnamaldehyde group were significantly lower (*P* < 0.01 or *P* < 0.05), but those in the cinnamaldehyde group were not significantly different from those in the model group (*P* > 0.05). See Fig. 2.

### Comparison of the cardiac weight indices of the rats in each group

Compared with that in the control group, the HWI was apparently greater in the model group (*P* < 0.01). Compared with that in the model group, the HWI was significantly lower in the propranolol and cinnamaldehyde high-dose groups (*P* < 0.01 or *P* < 0.05), but no significant decrease in the HWI was observed in the cinnamaldehyde low-dose group (*P* > 0.05). See Table 3.

**Table 3:** Heart weight ratio of rats in each group ($\overline{x} \pm s$)

Group	*n*	HWI
Control group	10	0.39 ± 0.04
Model group	8	0.49 ± 0.03^##^
Propranolol group	10	0.45 ± 0.04^**^
Cinnamaldehyde low-dose group	9	0.47 ± 0.03
Cinnamaldehyde high-dose group	10	0.45 ± 0.02^*^

I Control group, II Model group, III Propranolol group, IV Cinnamaldehyde low-dose group

V Cinnamaldehyde high-dose group

Note: vs Control group, ^##^*P* < 0.01; vs Model group, ^*^P < 0.05, ^**^P < 0.01

### Comparison of rat cardiomyocyte injury in each group

**Fig. 3:**
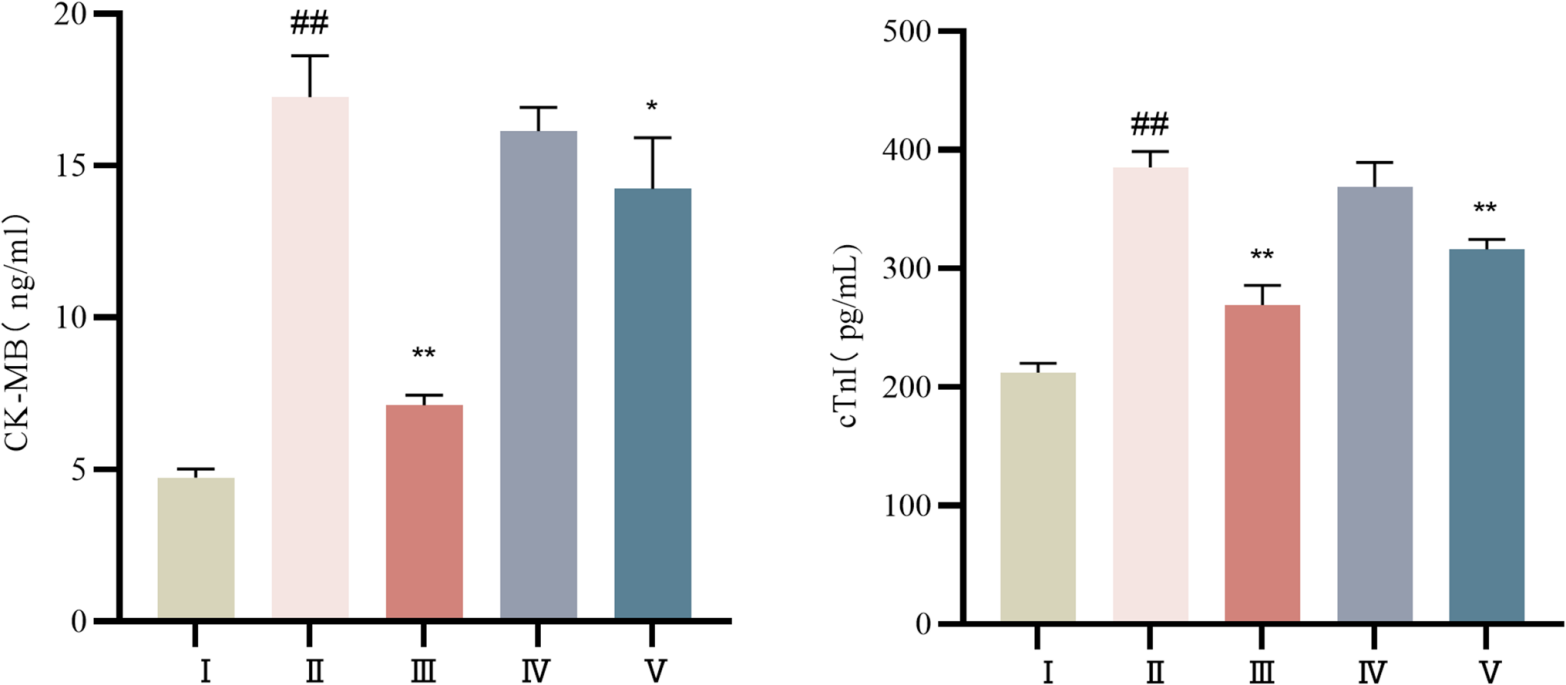
Levels of CK-MB and cTnI in each group. I Control group, II Model group, III Propranolol group, IV Cinnamaldehyde low dose group, V Cinnamaldehyde high-dose group. Note: vs Control group, ^##^*P* < 0.01; vs Model group, ^*^P < 0.05, ^**^P < 0.01

Compared with those in the control group, the CK-MB and cTnI levels in the serum of the rats in the model group were clearly elevated (*P* < 0.01). Compared with those in the model group, the serum cTnI and CK-MB levels were decreased in the propranolol and high-dose groups (*P* < 0.01 or *P* < 0.05). The serum CK-MB and cTnI levels in rats treated with low-dose cinnamaldehyde were also decreased. See Table 4 and Fig. 3 for details.

**Table 4:** Comparison of serum CK-MB and cTnI levels of rats in each group ($\overline{x} \pm s$)

Group	*n*	CK-MB(ng/mL)	cTnI(pg/mL)
Control group	10	4.74 ± 0.28	212.09 ± 8.33
Model group	8	17.26 ± 1.37^##^	383.40 ± 13.23^##^
Propranolol group	10	7.13 ± 0.34^**^	268.99 ± 16.65^**^
Cinnamaldehyde low dose group	9	16.15 ± 0.78	368.65 ± 20.76
Cinnamaldehyde high dose group	10	14.24 ± 1.68^*^	315.88 ± 8.67^**^

Note: vs Control group, ^##^*P* < 0.01; vs Model group, ^*^P < 0.05, ^**^P < 0.01

### Cardiac histopathology changes in the myocardium of rats in each group

**Fig. 4:**
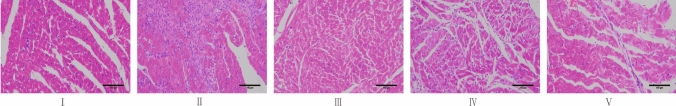
Pathological morphology of myocardial tissue of SD rats in each group (× 200). I Control group, II Model group, III Propranolol group, IV Cinnamaldehyde low dose group, V Cinnamaldehyde high-dose group

HE staining revealed that the cardiomyocytes of control rats were well-defined and well arranged with no inflammatory cell infiltration. In the model group, cardiomyocytes were unclear and disordered, with local cardiomyocyte lysis and necrosis and inflammatory cell infiltration. Compared with the model group, the myocardial cell structure in the low-dose cinnamaldehyde group was relatively clear, the cell arrangement was relatively neat, the myocardial cell dissolution and necrosis were reduced, and some inflammatory cells infiltrated. In the propranolol group and cinnamaldehyde high dose group, the myocardial cells were improved obviously, the cell structure was clear, the myocardial fibers were arranged neatly, and the local necrosis and inflammatory cell infiltration were significantly reduced. See Fig. 4.

### Comparison of mitochondrial injury in the cardiomyocytes of rats in each group

**Fig.5:**
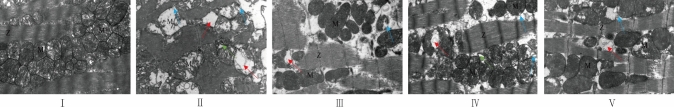
Comparison of myocardial mitochondrial damage in each group. I Control group, II Model group, III Propranolol group, IV Cinnamaldehyde low dose group, V Cinnamaldehyde high-dose group. Note: Red arrow: focal cavitation; Blue arrow: membrane damage; Green arrow: Ridge finger pattern change

Electron microscopy revealed that the cardiomyocytes of control rats had intact ultrastructures, and the myocardial myofibril structures were closely arranged with clear light and dark belts. The mitochondria were well arranged and evenly distributed, with intact mitochondrial envelopes and dense cristae. The model group showed severe damage to the ultrastructure, with extensive dissolution and thinning of the intracellular matrix, degradation and reduction of muscle fibres, and disordered structural arrangement of myofibers and filaments. Decreases in the number of mitochondria, uneven distribution, obvious swelling, uneven size, partial membrane damage, massive fracture of cristae, shortening or dissolution, matrix dissolution, and partial focal cavitation were observed. In the Western medicine group, some mitochondria were slightly swollen and increased in volume, and vacuolation was rare. The damage to the myocardial structure in the low-dose cinnamaldehyde group was similar to that in the model group, but the light and dark bands were clear. The high-dose cinnamaldehyde group was significantly improved, but some mitochondria were still slightly swollen, and vacuoles were rare. See Fig. 5.

### Comparison of ROS levels in the myocardial tissue of rats in each group

**Fig.6:**
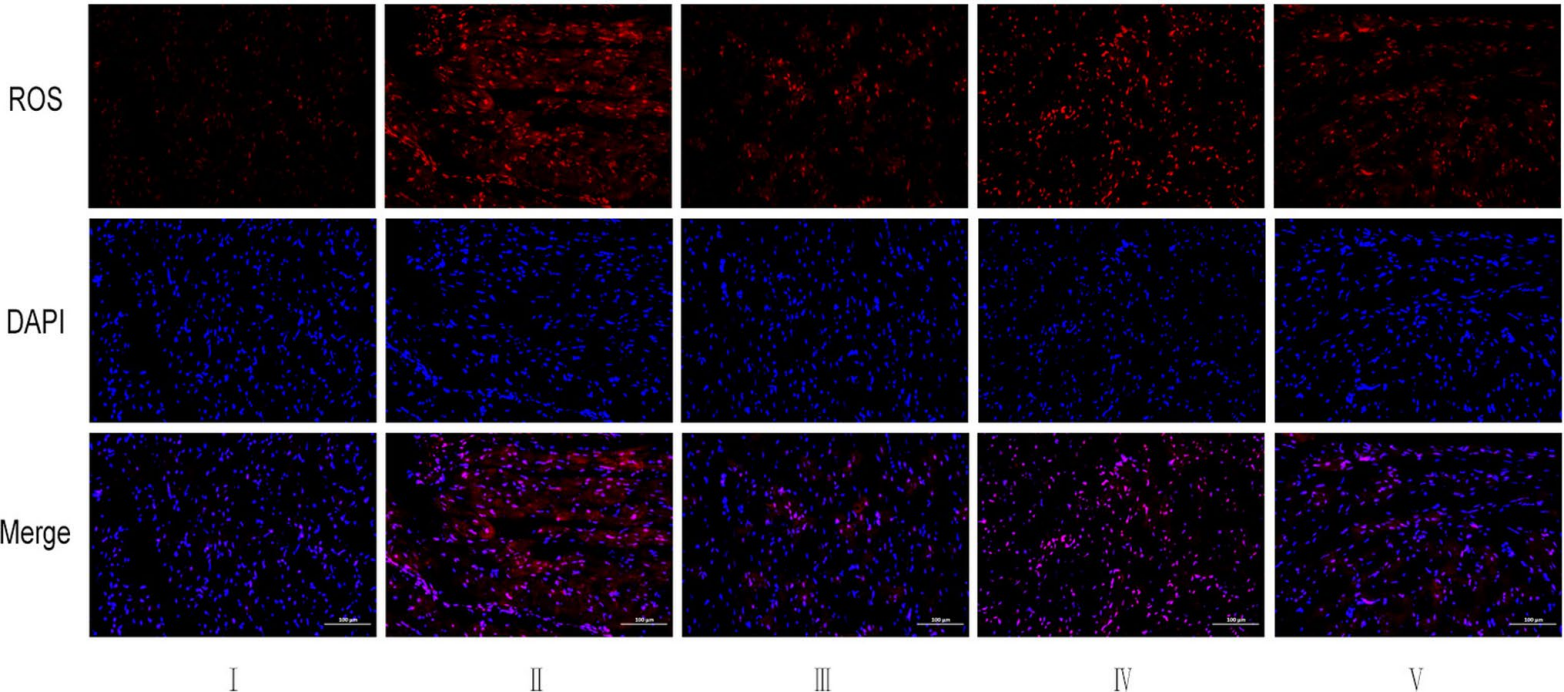
ROS immunofluorescence staining results of myocardial tissue of rats in each group(× 200). I Control group, II Model group, III Propranolol group, IV Cinnamaldehyde low dose group, V Cinnamaldehyde high-dose group

Immunofluorescence revealed that red fluorescence was not evident in the cardiac tissue of control rats, indicating low ROS levels in the myocardial tissue. Compared with that in the control group, the red fluorescence in the model group was increased, suggesting increased ROS levels in the myocardial tissue. Compared with the model group, the fluorescence intensity of ROS in myocardial tissue of low dose cinnamaldehyde group was not significantly decreased, while that of the propranolol group and high-dose cinnamaldehyde group was significantly decreased, indicating that propranolol and cinnamaldehyde treatment can reduce the ROS level in myocardial tissue of ventricular arrhythmia rats. See Fig. 6.

### Protein expression levels of TAK1 and p-TAK1 in the myocardial tissue of rats in each group

#### The protein expression level of p-TAK1 was determined by the SABC method

**Fig.7:**
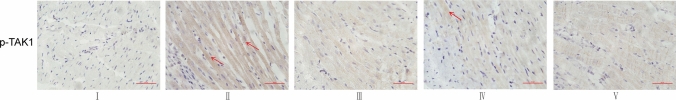
Effect of cinnamaldehyde on p-TAK1 protein expression in myocardial tissue of SD rats in each group (× 400). I Control group, II Model group, III Propranolol group, IV Cinnamaldehyde low dose group, V Cinnamaldehyde high-dose group

Immunohistochemistry revealed that p-TAK1 protein expression in the myocardial tissue of the control group was very low. Compared with that in the control group, p-TAK1 protein expression was significantly increased in the model group, and p-TAK1 was mainly expressed in the cytoplasm. Compared with that in the model group, p-TAK1 protein expression was significantly downregulated in the propranolol and high-dose groups of cinnamaldehyde; however, this reduction was not obvious in the low-dose group of cinnamaldehyde. See Fig. 7.

#### The protein expression levels of p-TAK1 and TAK1 were determined by Western blot

**Fig.8:**
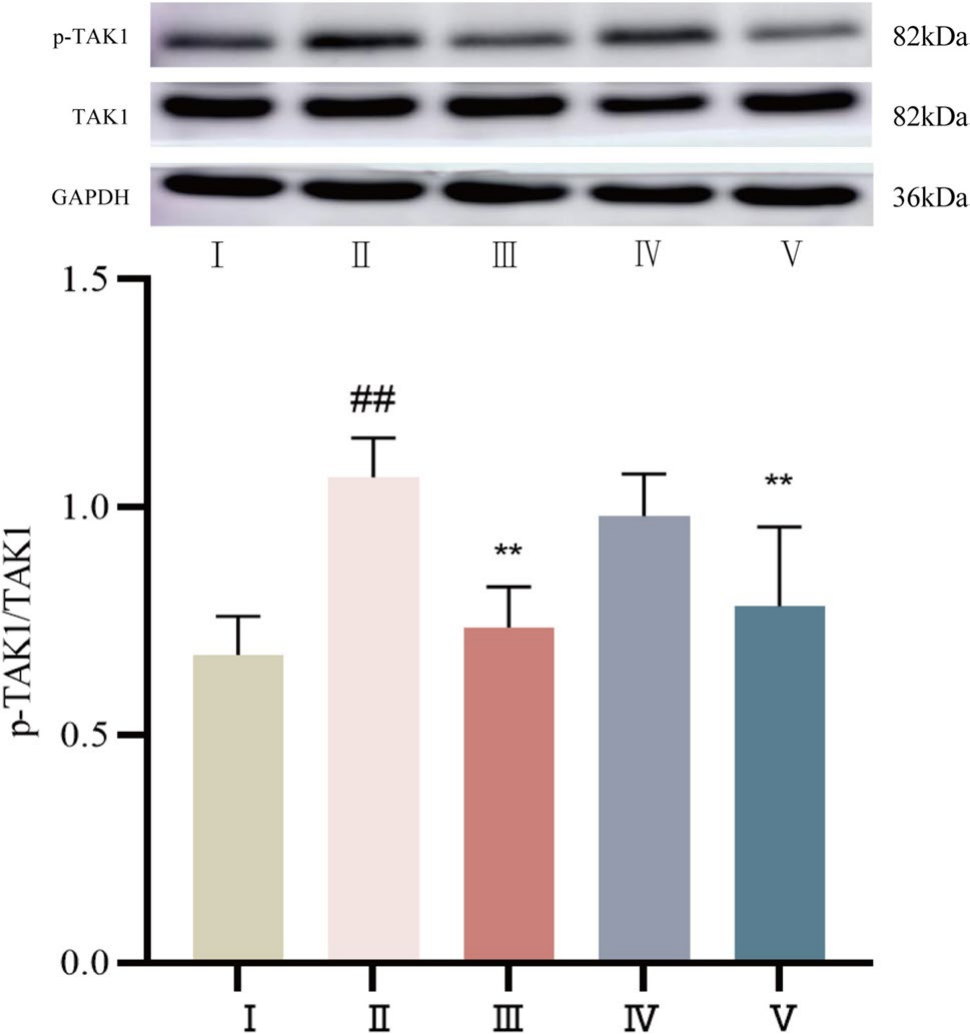
The expression of p-TAK1 protein in myocardial tissue of SD rats in each group. I Control group, II Model group, III Propranolol group, IV Cinnamaldehyde low dose group, V Cinnamaldehyde high-dose group. Note: vs Control group, ^##^*P* < 0.01; vs Model group, ^**^P < 0.01

Compared with that in the control group, p-TAK1 protein expression was significantly increased in the myocardial tissue of rats in the model group (*P* < 0.01). Compared with that in the model group, p-TAK1 protein expression was decreased in both the propranolol and cinnamaldehyde high-dose groups (*P* < 0.01). The protein expression of p-TAK1 was slightly reduced in the low-dose cinnamaldehyde group (*P* > 0.05). See Fig. 8.

### Comparison of the protein expression levels of p38MAPK and p-p38MAPK in each group

#### The protein expression level of p-p38MAPK was determined by the SABC method

**Fig.9:**
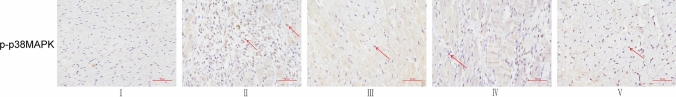
Effect of cinnamaldehyde on p-p38MAPK protein expression in myocardial tissue of SD rats in each group (× 400). I Control group, II Model group, III Propranolol group, IV Cinnamaldehyde low dose group, V Cinnamaldehyde high-dose group

p-p38MAPK is expressed mainly in the cell nucleus. Immunohistochemistry showed that the control group exhibited weak p-p38MAPK expression. Compared with that in the control group, the protein expression of p-p38MAPK in the SD group decreased, and the protein expression of p-p38MAPK decreased after the administration of propranolol and cinnamaldehyde. See Fig. 9.

#### The protein expression levels of p-p38MAPK and p38MAPK were detected by Western blot

**Fig.10:**
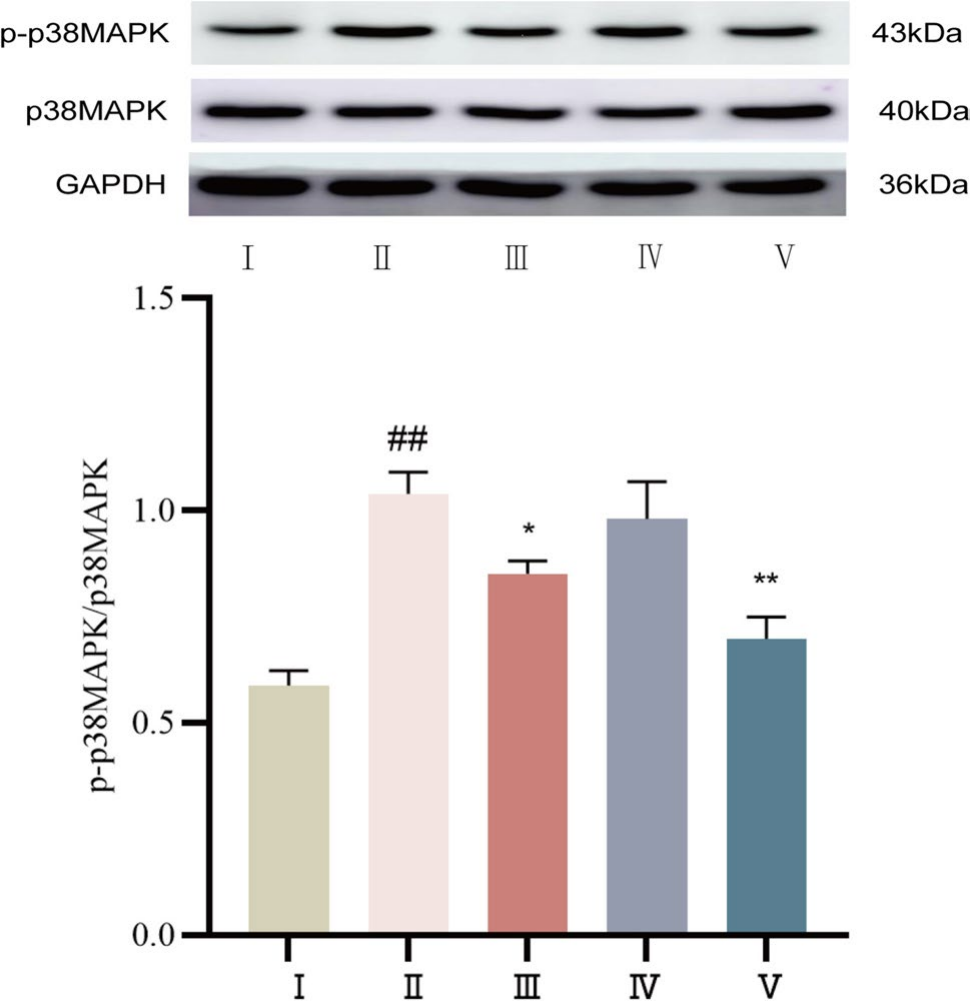
Expression of p-p38MAPK protein in myocardial tissue of SD rats in each group. I Control group, II Model group, III Propranolol group, IV Cinnamaldehyde low dose group, V Cinnamaldehyde high-dose group. Note: vs Control group, ^##^*P* < 0.01; vs Model group, ^*^P < 0.05, ^**^P < 0.01

The Western blot results showed that the control group exhibited weak p-p38MAPK expression. Compared with that in the control group, p-p38MAPK protein expression in the model group of SD rats was significantly greater (*P* < 0.01). Compared with that in the model group, p-p38MAPK was decreased in the propranolol group and cinnamaldehyde high-dose group (*P* < 0.01 or *P* < 0.05), but its expression was not downregulated in the low-dose group (*P* > 0.05). See Fig. 10.

### Comparison of the protein expression levels of p-NF-κB and NF-κB in the myocardial tissue of each rat

#### The protein expression level of p-NF-κB was determined by the SABC method

**Fig.11:**
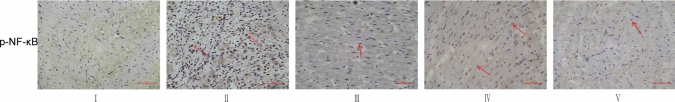
Effect of cinnamaldehyde on p-NF-κB protein expression in myocardial tissue of SD rats in each group (× 400). I Control group, II Model group, III Propranolol group, IV Cinnamaldehyde low dose group, V Cinnamaldehyde high-dose group

Immunohistochemistry revealed that there was almost no p-NF-κB in the mycelial tissue of the control group. Compared with that in the control group, the expression of p-NF-κB, which is mainly expressed in the nucleus, was significantly increased in the model group. Compared to that in the model group, the protein expression of p-NF-κB was somewhat downregulated in the three treatment groups. See Fig. 11.

#### The protein expression levels of p-NF-κB and NF-κB were determined by Western blot

**Fig.12:**
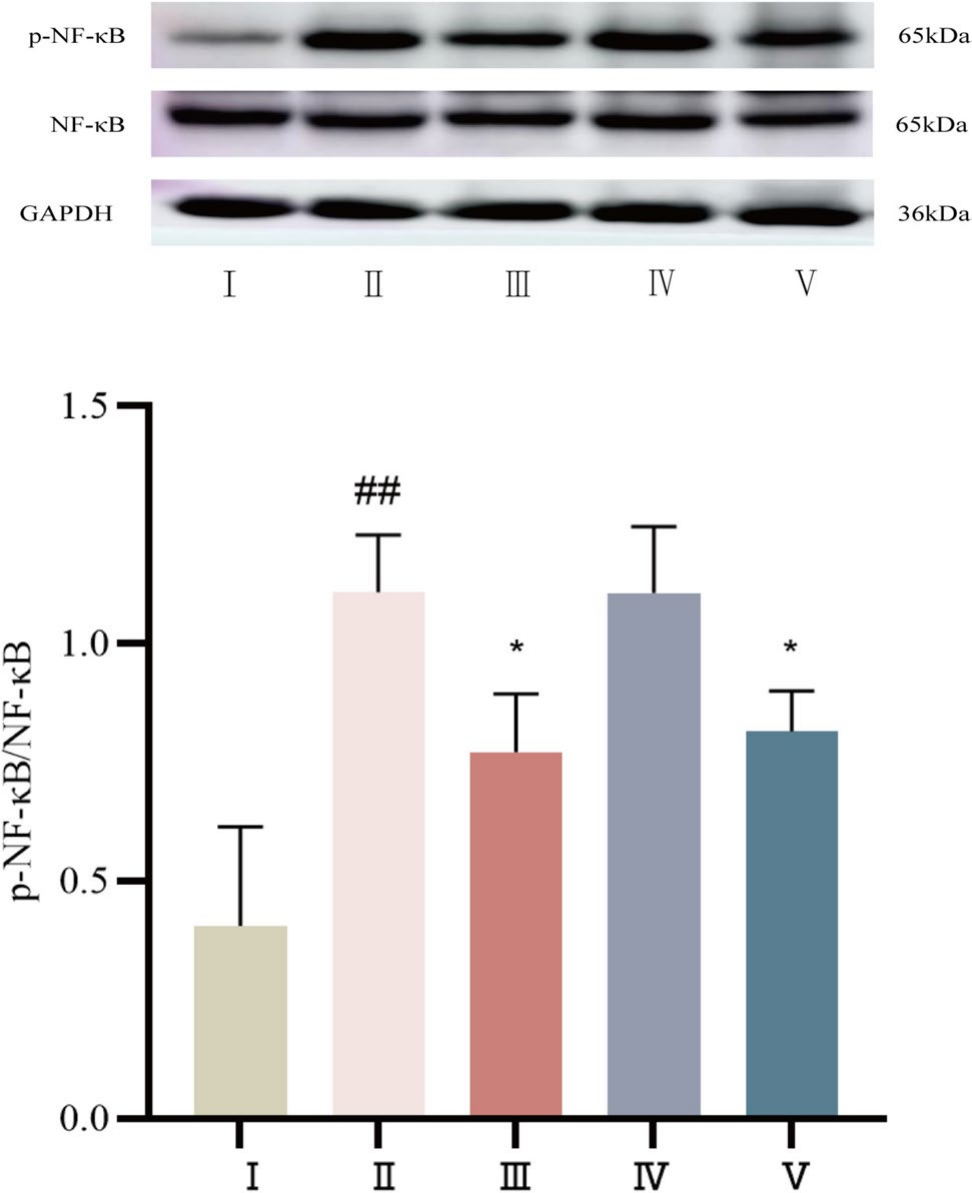
Expression of p-NF-κB protein in myocardial tissue of SD rats in each group. I Control group, II Model group, III Propranolol group, IV Cinnamaldehyde low dose group, V Cinnamaldehyde high-dose group. Note: vs Control group, ^##^*P* < 0.01; vs Model group, ^*^P < 0.05

The myocardial tissue of control SD rats expressed less p-NF-κB protein. Compared with that in the control group, p-NF-κB protein expression was significantly greater (*P* < 0.01). Compared with that in the model group, p-NF-κB protein expression was decreased in both the propranolol and cinnamaldehyde high-dose groups (*P* < 0.05). See Fig. 12.

### Comparison of NLRP3 gene and protein expression levels in myocardial tissues of rats

#### The mRNA expression levels of NLRP3 were determined by RT‒PCR

**Fig.13:**
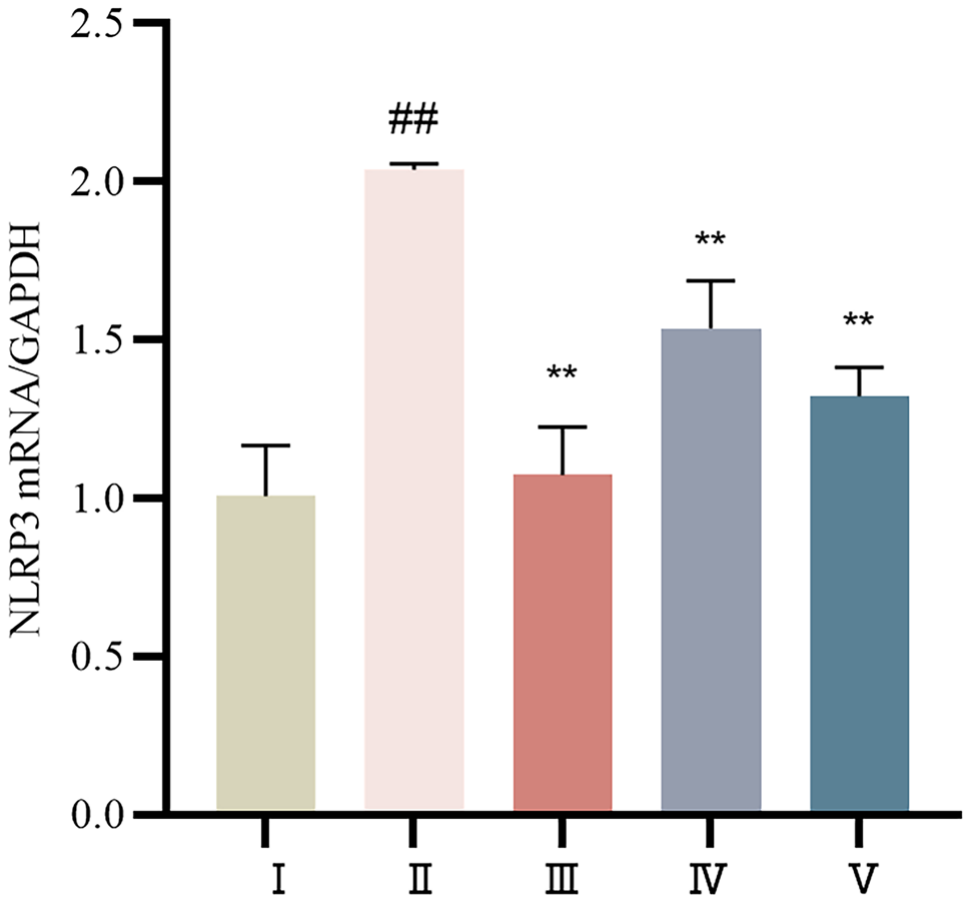
Comparison of NLRP3 mRNA expression in myocardial tissue of SD rats in each group was shown in the histogram. I Control group, II Model group, III Propranolol group, IV Cinnamaldehyde low dose group, V Cinnamaldehyde high-dose group. Note: vs Control group, ^##^*P* < 0.01; vs Model group, ^**^P < 0.01

Compared with those in the control group, the expression levels of myocardial NLRP3 mRNA in the model group were significantly greater (*P* < 0.01). Compared with those in the model group, the expression levels in both the propranolol and cinnamaldehyde groups were lower (*P* < 0.01). The detailed results are shown in Table 5 and Fig. 13.

**Table 5:** Comparison of NLRP3 mRNA expression levels in myocardial tissue of SD rats in each group ($\overline{x} \pm s$)

Group	*n*	NLRP3 mRNA	IL-1β mRNA
Control group	10	1.01 ± 0.158	1.00 ± 0.07
Model group	8	2.04 ± 0.019^##^	3.08 ± 0.34^#^
Propranolol group	10	1.07 ± 0.15^**^	1.26 ± 0.12^**^
Cinnamaldehyde low-dose group	9	1.54 ± 0.15^**^	2.40 ± 0.52
Cinnamaldehyde high-dose group	10	1.32 ± 0.092^**^	1.16 ± 0.15^**^

Note: vs Control group, ^##^*P* < 0.01; vs Model group, ^**^P < 0.01

#### NLRP3 protein expression levels were determined by the SABC method

**Fig.14:**
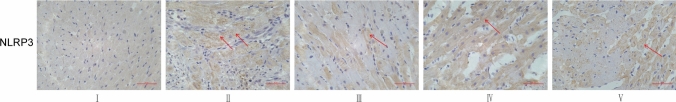
Effect of cinnamaldehyde on NLRP3 protein expression in myocardial tissue of SD rats in each group (× 400). I Control group, II Model group, III Propranolol group, IV Cinnamaldehyde low dose group, V Cinnamaldehyde high-dose group

NLRP3 in the model group was mainly expressed in the cytoplasm. NLRP3 protein expression was significantly greater in the model group than in the control group. Compared with that in the model group, NLRP3 protein expression was downregulated in the three treatment groups and more pronounced in the propranolol group and the cinnamaldehyde high-dose group. See Fig. 14.

#### The NLRP3 protein expression level was determined by Western blot

**Fig.15:**
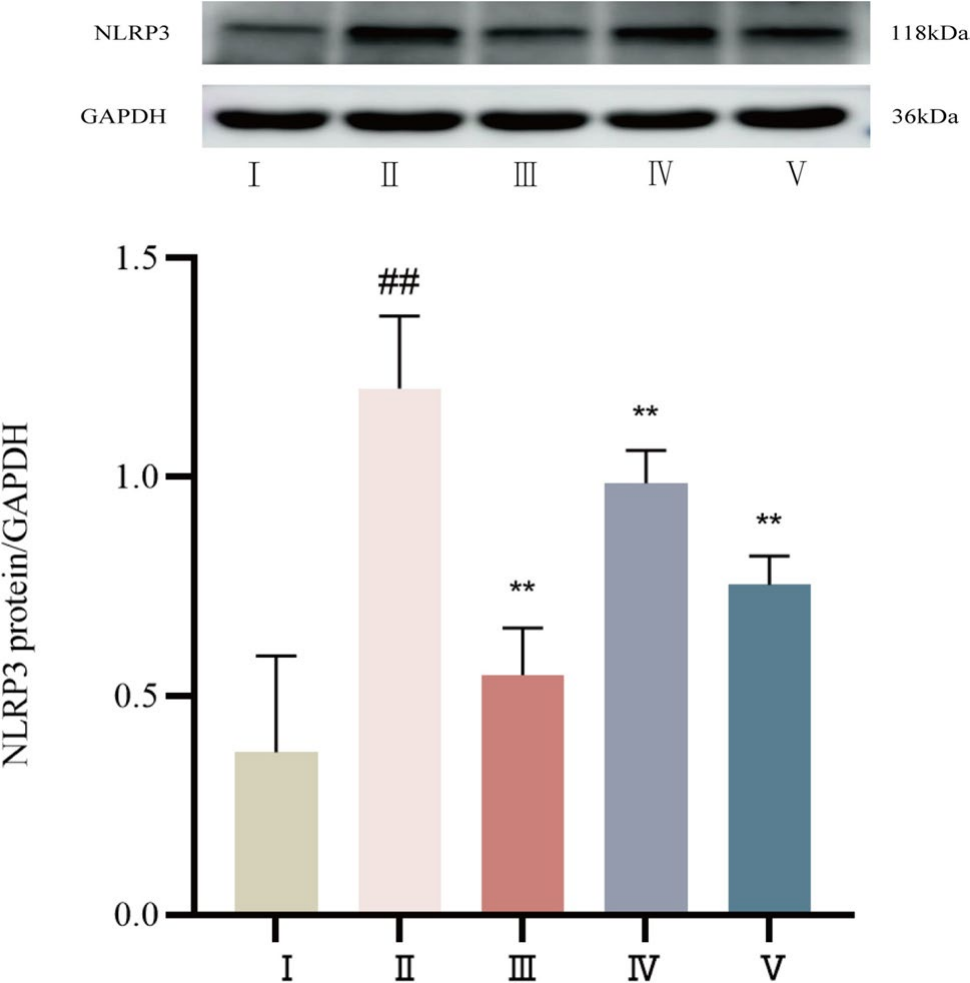
NLRP3 protein expression in myocardial tissue of SD rats in each group. I Control group, II Model group, III Propranolol group, IV Cinnamaldehyde low dose group, V Cinnamaldehyde high-dose group. Note: vs Control group, ^##^*P* < 0.01; vs Model group, ^**^P < 0.01

The Western blot results showed that control rats had decreased NLRP3 protein expression in myocardial tissue. NLRP3 protein expression was significantly greater in the model group than in the control group (*P* < 0.01). Compared with that in the model group, NLRP3 protein expression was significantly lower in both the propranolol and cinnamaldehyde groups (*P* < 0.01). See Fig. 15.

### Comparison of the IL-1β levels in the rats in each group

#### Comparison of IL-1β levels in the serum of each group

**Fig.16:**
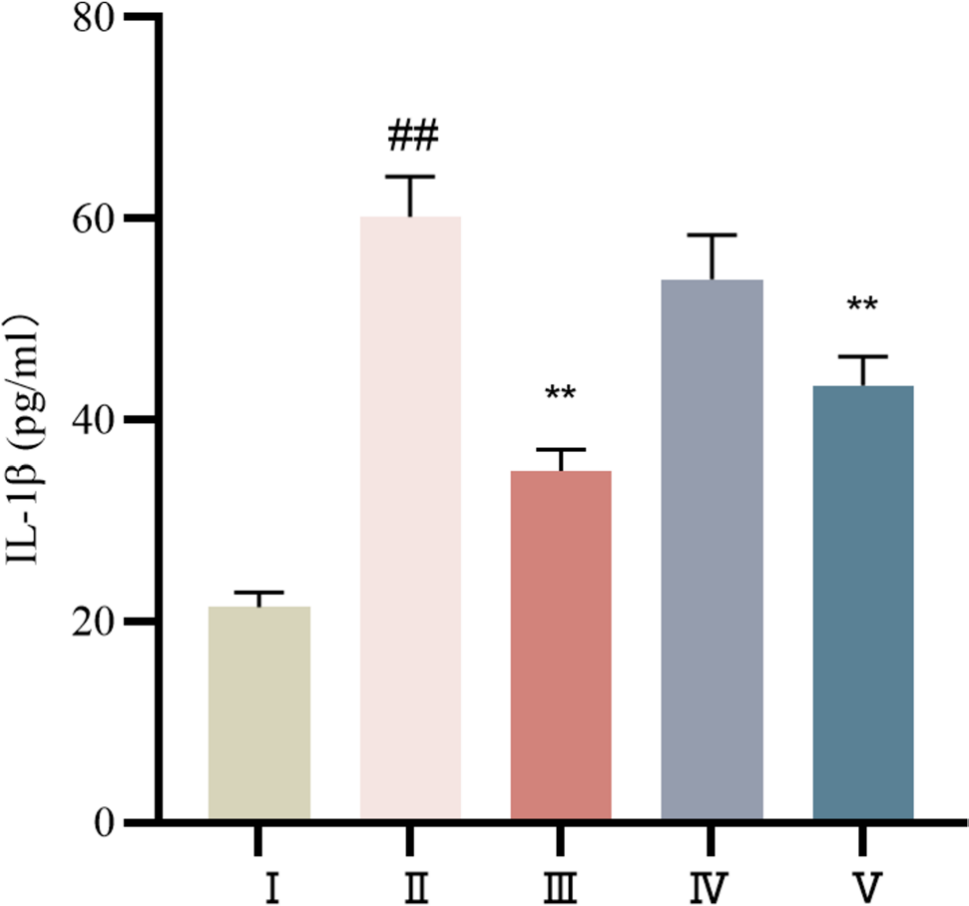
Serum IL-1β levels of rats in each group. I Control group, II Model group, III Propranolol group, IV Cinnamaldehyde low dose group, V Cinnamaldehyde high-dose group. Note: vs Control group, ^##^*P* < 0.01; vs Model group, ^**^P < 0.01

Compared with those in the control group, the serum IL-1β levels in the model group were significantly greater (*P* < 0.01), and the serum IL-1β levels were significantly lower (*P* < 0.01). Compared to those in the model group, the serum IL-1β levels in both the propranolol and cinnamaldehyde high-dose groups were significantly lower. The IL-1β level was also lower in the low-dose cinnamaldehyde group, but the difference was not statistically significant (*P* > 0.05). See Table 6 and Fig. 16 for more details.

**Table 6:** Comparison of serum IL-1β content of rats in each group ($\overline{x} \pm s$)

Group	*n*	IL-1β(pg/mL)
Control group	10	21.44 ± 1.44
Model group	8	60.17 ± 3.97^##^
Propranolol group	10	34.94 ± 2.09^**^
Cinnamaldehyde low dose group	9	53.97 ± 4.39
Cinnamaldehyde high dose group	10	43.46 ± 2.82^**^

Note: vs Control group, ^##^*P* < 0.1; vs Model group, ^**^P < 0.01

#### mRNA expression levels of IL-1β in the myocardial tissues of rats in each group

**Fig.17:**
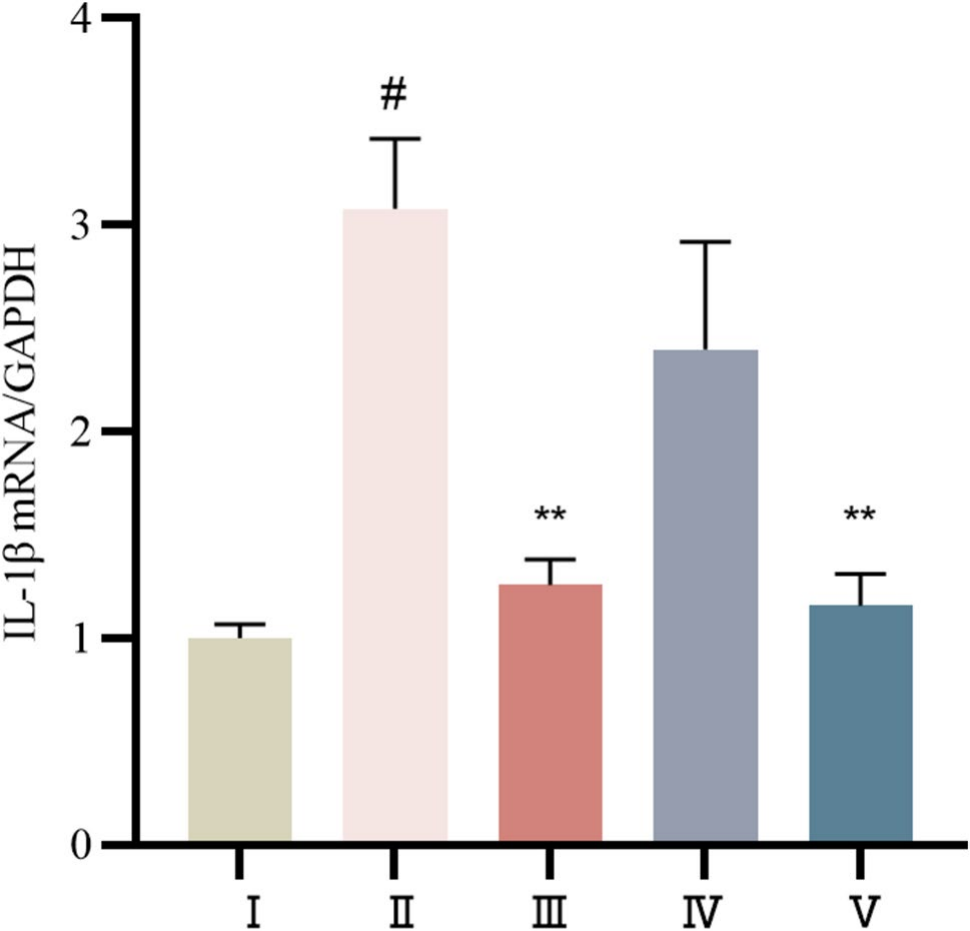
mRNA levels of IL-1β in myocardium of rats in each group. I Control group, II Model group, III Propranolol group, IV Cinnamaldehyde low dose group, V Cinnamaldehyde high-dose group. Note: vs Control group, ^#^*P* < 0.05; vs Model group, ^**^P < 0.01

Compared with those in the control group, the gene expression levels of IL-1β in the rats were significantly greater(*P* < 0.01). Compared with those in the model group, the gene expression levels of IL-1β were reduced in both the propranolol and cinnamaldehyde groups, and the downregulation of IL-1β was more pronounced in the propranolol group and the cinnamaldehyde high-dose group(*P* < 0.01). See Fig. 17.

#### The IL-1β protein expression level was measured by the SABC method

**Fig.18:**
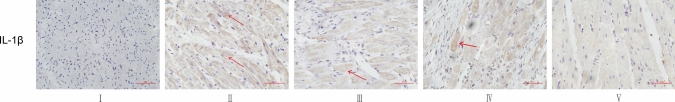
Effect of cinnamaldehyde on IL-1β protein expression in myocardial tissue of SD rats in each group (× 400). I Control group, II Model group, III Propranolol group, IV Cinnamaldehyde low dose group, V Cinnamaldehyde high-dose group

The immunohistochemistry results showed that IL-1β expression in the control group was not obvious, and IL-1β protein expression was significantly increased in the model group, mainly in the cytoplasm. Compared with that in the model group, the IL-1β expression in the three treatment groups decreased to different degrees. See Fig. 18.

#### The protein expression level of IL-1β was determined by Western blot

**Fig.19:**
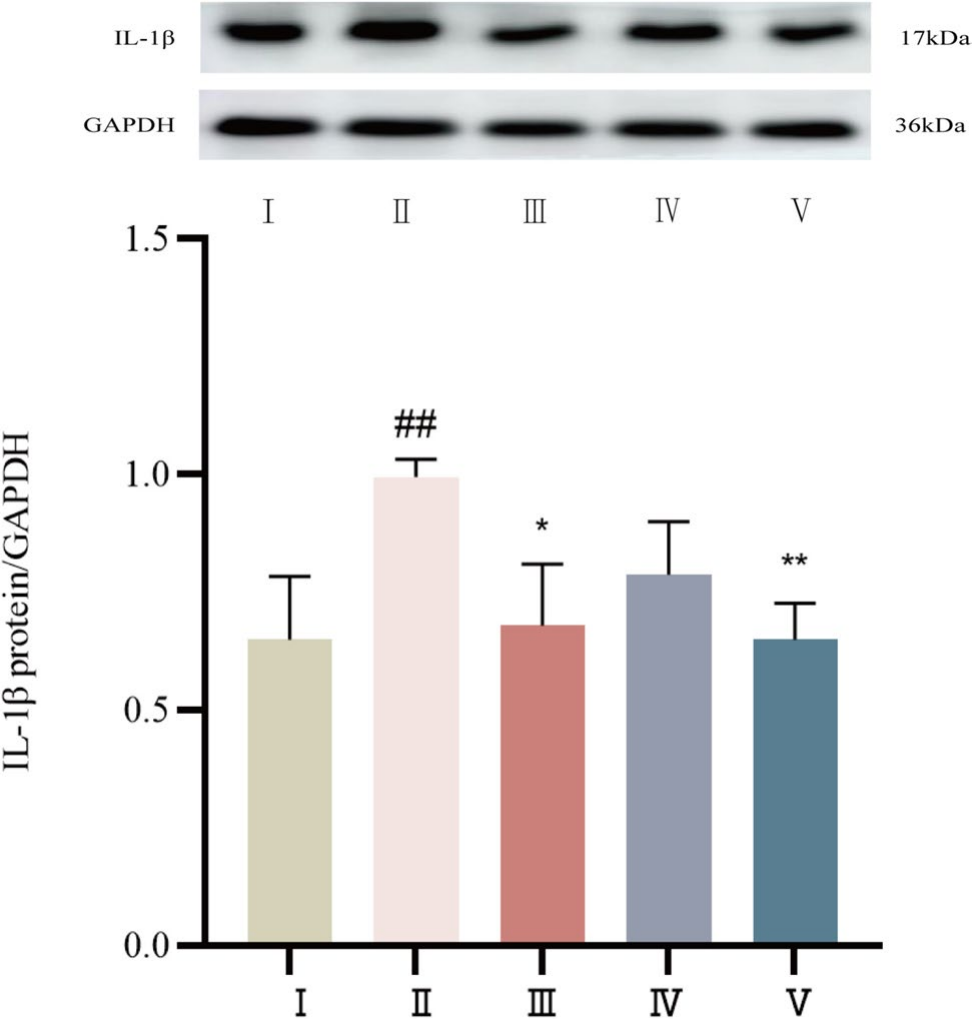
Expression of IL-1β protein in myocardial tissue of SD rats in each group. I Control group, II Model group, III Propranolol group, IV Cinnamaldehyde low dose group, V Cinnamaldehyde high-dose group. Note: vs Control group ^##^*P* < 0.01; vs Model group, ^*^P < 0.05, ^**^P < 0.01

IL-1β expression was weak in the control group. Compared with that in the control group, IL-1β expression was significantly increased in the model group (*P* < 0.01). Compared with that in the model group, IL-1β expression was significantly attenuated in the propranolol group and the cinnamaldehyde high-dose group (*P* < 0.01 or *P* < 0.05), and that in the cinnamaldehyde low-dose group also showed a decreasing trend (*P* > 0.05). See Fig. 19.

## Discussion

Sudden cardiac death is the leading cause of death in CVD patients, while 85% of sudden cardiac deaths originate from ventricular arrhythmia [19]. With the development of radiofrequency ablation technology and the application of implantable defibrillation cardioverter, the risk of sudden cardiac death caused by ventricular arrhythmia has been reduced, but due to technical limitations, high price and regional differences, drug therapy is still the main treatment for ventricular arrhythmia. At present, βblockers, sodium channel blockers, potassium channel blockers and non-dihydropyridine calcium antagonists are commonly used in clinics to prevent and treat ventricular arrhythmia. However, due to the complexity and individualization of the disease, some patients have not achieved significant curative effects after the above-mentioned drug treatment. Therefore, it is urgent to find the pathogenesis of ventricular arrhythmia and explore new therapeutic drug targets. The pathogenesis of ventricular arrhythmia has not been fully elucidated, and previous research has shown that the inflammatory response and oxidative stress are involved in the process of ventricular arrhythmia disease [4]. We speculate that the inflammatory response interacts with oxidative stress to activate TAK1, which activates downstream p38MAPK molecules through a phosphorylation cascade, and after p38MAPK activation, which acts on the corresponding substrate, further activates the NLRP3 inflammasome. Pyroptosis is a mode of inflammatory cell death that can increase proinflammatory cytokine release and accelerate the disease process [18, 20]. Therefore, based on a previous study, this study further explored the inflammatory damage mechanism of ventricular arrhythmia and detected the expression of key molecules of the TAK1-p38MAPK-NLRP3 pathway in the myocardial tissue of ventricular arrhythmia model rats. Propranolol was selected as a positive control because it blocks the cardiomyocyte surface β receptor, antagonizes sympathetic excitation and reduces catecholamine release, thus reducing susceptibility to arrhythmia [21, 22]. Cinnamaldehyde, the main active component of Cassia twigs, has strong antioxidant and anti-inflammatory activities and can significantly improve cardiac function and effectively antagonize ischaemic arrhythmia [4].

### Reducing ISO-induced myocardial injury is a key mechanism by which cinnamaldehyde protects against ventricular arrhythmia

ISO is a β-adrenocellular agonist that can excite β receptors on the surface of cardiomyocytes, increase heart rate, accelerate conduction, enhance myocardial contraction, promote myocardial ischaemia and hypoxia, and subsequently cause myocardial injury. Manolis A et al. reported that myocardial injury can easily cause increased release of catecholamine substances and scars, thus affecting myocardial electrophysiological signalling and promoting the development of ventricular arrhythmias [4, 23, 24]. Bostan et al. [25] showed that cTnI and CK-MB are the key active substances involved in cardiomyocyte contraction. After the integrity and permeability of the cardiomyocyte membrane are disrupted, cardiomyocytes escape from myocardial tissue to the blood, resulting in abnormal increases in cTnI and CK-MB in the serum, and their levels are positively correlated with the degree of cardiomyocyte damage. The HWI can also reflect the degree of myocardial damage to some extent. ISO injection leads to an increase in blood pressure and cardiac load in SD rats and eventually leads to compensatory hypertrophy of the heart, while myocardial oxygen demand increases, which exacerbates ischaemia and further aggravates injury [4]. The results of this study showed that the serum cTnI, CK-MB and HWI increased significantly in SD rats induced by ISO, which was consistent with the results of Feriani et al. [26]. Different degrees of ventricular arrhythmia, especially SVT, occurred in the model group, with increased arrhythmia scores. High-dose cinnamaldehyde reduces the serum cTnI and CK-MB levels, improves cardiac hypertrophy, and subsequently reduces the SVT to attenuate the severity of ventricular arrhythmia and reduce the arrhythmia score. Therefore, the findings of the present study suggested that the mechanism by which cinnamaldehyde protects against ventricular arrhythmia may be strongly related to its ability to effectively reduce cardiomyocyte injury.

### Cinnamaldehyde alleviates myocardial injury in rats with ventricular arrhythmia by inhibiting inflammatory responses

Previous studies have shown that inflammatory myocardial injury is a key factor in promoting the development of ventricular arrhythmias. During inflammation, inflammatory cell aggregation and inflammatory cytokine release are able to participate in the processes of myocardial ischaemia, hypertrophy, and fibrosis, causing severe myocardial tissue damage and increasing susceptibility to ventricular arrhythmogenesis [27]. Jghef et al. confirmed that excessive release of proinflammatory cytokines, including IL-6, IL-1β, TGF-β1 and TNF-α, can occur in ISO-induced acute myocardial infarction, myocardial fibrosis and heart failure; among these cytokines, IL-1β is the most critical inflammatory mediator, causing neutrophils and macrophages to accumulate in the damaged myocardium, further promoting the release of more inflammatory cytokines and aggravating myocardial inflammatory injury [28–30]. As shown in the present study, the cardiomyocytes of ventricular arrhythmia rats exhibited a disordered arrangement, local cell lysis and necrosis, and increased inflammatory cell infiltration; moreover, the serum and myocardial tissue IL-1β expression levels increased, leading to an inflammatory response. Treatment with a high dose reversed the above changes, indicating that cinnamaldehyde may play a protective role by inhibiting the inflammatory response.

### Cinnamaldehyde alleviates inflammatory injury in the myocardium of ventricular arrhythmia rats by inhibiting NLRP3-mediated cardiomyocyte inflammatory factor released

As reported by Kinra et al. [31] Pathogen-associated molecular patterns(PAMPs) or damage-associated molecular patterns(DAMPs) and Toll-like receptors(TLRs) combine and activate NF-κB to form the first signal of NLRP3 inflammasome activation, initiating the expression of the precursor of NLRP3(caspase-1, IL-1β and IL-18) and completing the quantitative accumulation of inflammasome-related molecules. Furthermore, NF-κB stimulates qualitative NLRP3 activation through the recruitment of ASC and caspase-1 precursors to form inflammasomes, and caspase-1 self-shear activation can reduce the precursor of IL-1β and IL-18, promote the generation of active IL-1β and IL-18, participate in inflammatory reaction and aggravate the inflammatory injury of myocardial cells. In this study, RT‒PCR and Western blotting revealed that cinnamaldehyde reduced the gene and protein expression of NLRP3 in the myocardium of rats with ventricular arrhythmia, thus reducing the release of inflammatory factors and reducing inflammatory injury to the myocardium. At the same time, considering that the activation of NLRP3 inflammasome, whose activation mediates pyroptosis [31], this study further speculated that cinnamaldehyde may alleviate the myocarditis injury of ventricular arrhythmia rats by inhibiting the release of inflammatory factors caused by NLRP3-mediated myocardial pyroptosis.

### Cinnamaldehyde inhibited rat cardiac NLRP3 expression by regulating the TAK1-p38MAPK/NF-κB pathway

Mitogen-activated protein kinases(MAPKs) are a group of serine/threonine protein kinases that, through the "MAPKKK → MAPKK → MAPK" tertiary cascade process, mediate the transduction of extracellular signals from the cell membrane to the nucleus and regulate cell differentiation, apoptosis, inflammation and other pathophysiological processes. TAK1 is a MAPKKK that can be activated by hypoxia and inflammation [32]. After the activation of TAK1 phosphorylation, TAK1 continues to transduce downstream signals and ultimately acts on the threonine and tyrosine duplex of p38MAPK, subsequently activating it. Consistent with the findings of Guo et al. [33] in this study, p-TAK1 and p-p38MAPK expression were significantly increased in the myocardial tissues of rats in the model group. Zhao et al. [34] reported that TAK1-p38MAPK signalling functions by activating NF-κB; that is, activated p38MAPK translocates to the nucleus and further phosphorylates the transcription factor NF-κB, and activated NF-κB provides the first signal for NLRP3 inflammasome activation, promotes pyroptosis, and regulates the overexpression of the inflammatory factors IL-1β and IL-18. Xu et al. [5] showed that mature IL-1β, IL-18 and other DAMPs are released into the extracellular space, which further activates TAK1 to initiate a new round of TAK1-p38MAPK-NLRP3 inflammatory pathway activation, expands the inflammatory response, exacerbate inflammatory damage in the heart, and induce severe ventricular arrhythmia. In this study, SABC and Western blot analyses also verified that the protein expression of p-NF-κB increased after ISO intervention. However, cinnamaldehyde treatment significantly reduced the protein expression of p-TAK1, p-p38MAPK and p-NF-κB in the myocardium of rats with ventricular arrhythmias, which indicates that by regulating the TAK1 signalling cascade, cinnamaldehyde downregulates p-NF-κB expression, suppresses NLRP3 activation and protects ventricular arrhythmia rats from myocardial injury.

### 4.5 Inhibition of oxidative stress is an important mechanism by which cinnamaldehyde interferes with the TAK1 signalling cascade and inflammatory injury in ventricular arrhythmia models

Oxidative stress refers to the cellular stress response caused by the excessive production of ROS in the body and an imbalance in the antioxidant system when the body is exposed to various harmful stimuli or needs to remove ageing cells from the body. The generation of ROS is mainly derived from mitochondria, and cardiomyocytes contain a large number of mitochondria; thus, cardiomyocytes are highly sensitive to oxidative stress [35]. Belosludtseva et al. [36] reported that in a model of ISO-induced acute myocardial injury, transmission electron microscopy revealed a significant disruption of mitochondrial integrity in myocardial tissue, releasing a large amount of ROS and causing oxidative myocardial damage. In this study, we found that in the model group, the mitochondria exhibited disorder, obvious swelling, vacuolation, partial membrane damage and crest fracture. Moreover, immunofluorescence showed an abnormal increase in myocardial ROS levels, and these findings are consistent with previous studies. Abdelsalam et al. [37] demonstrated that ROS can bind to TLR4 and then recruit myeloid differentiation Factor 88(MyD88). MyD88 transduces signals and activates the TAK1 signalling cascade, leading to cardiomyocyte injury. In addition, DAMPs such as ROS provide a second signal for NLRP3 inflammasome activation. Cinnamaldehyde exists in natural Cinnamomum plants, and Cinnamomum cassia is widely used in the prevention and treatment of cardiovascular diseases in Chinese traditional medicine. In modern pharmacology, cinnamaldehyde, as an effective component of Cinnamomum cassia, has attracted people's attention because of its antioxidant activity. A large number of literatures reported that cinnamaldehyde pretreatment can increase SOD activity and decrease MDA level in the myocardium [12, 38]. This study revealed that after high-dose cinnamaldehyde treatment, mitochondrial swelling in the myocardium of SD rats with ventricular arrhythmia improved ridge rupture, reduced ROS release, and inhibited TAK1 cascade activation and NLRP3 activation. Experiments have shown that the inhibition of oxidative stress then regulates TAK1-p38MAPK/NF-κB-NLRP3 pathway activation and that the alleviation of inflammation may be the key mechanism of action against ventricular arrhythmia.

In conclusion, the expression of the TAK1-p38MAPK-NLRP3 signalling pathway is increased in the myocardium of ventricular arrhythmic SD rats by improving mitochondrial dysfunction and reducing ROS generation. Cinnamaldehyde regulates the activation of this signalling pathway, inhibits cardiomyocyte pyroptosis, and antagonizes myocardial inflammatory injury, thus delaying the progression of the disease course. Studies have shown that cinnamaldehyde has a potential therapeutic effect on ventricular arrhythmias, and TAK1, p38MAPK and NLRP3 may be targets for its key effects. In the future, the expression of ASC, caspase-1 and IL-18 in the myocardium of ventricular arrhythmia rats will continue to increase, providing a theoretical basis for the resistance of cinnamaldehyde against myocardial inflammatory injury.
